# The effect of corrective exercises on musculoskeletal disorders among the older adults residing in a nursing home in Rasht, Guilan, Iran

**DOI:** 10.1186/s12891-023-06915-8

**Published:** 2023-10-17

**Authors:** Hamed Asadi, Azar Darvishpour, Kamran Ezzati, Bahare Gholami-chaboki

**Affiliations:** 1 Department of nursing, Zeyinab (P.B.U.H) School of Nursing and Midwifery, Guilan University of Medical Sciences, Rasht, Iran; 2 Social Determinants of Health (SDH) Research Center, Guilan University of Medical Sciences, Rasht, Iran; 3 Neuroscience Research Center, Poorsina Hospital, Faculty of medicine, Guilan University of Medical Sciences, Rasht, Iran; 4 Cardiovascular Research Center, Guilan University of Medical Sciences, Rasht, Iran

**Keywords:** Aging, Exercise Therapy, Musculoskeletal Diseases, Pain

## Abstract

**Background:**

Ageing causes changes in the function of musculoskeletal systems and disability, and injury among older adults. This study aimed to determine the effect of corrective exercises on musculoskeletal disorders among older adults residing in a nursing home in Iran.

**Method:**

This controlled clinical trial study was conducted on 58 older adults (29 samples in each group). The intervention group performed corrective exercises for 8 weeks and three sessions per week and each session lasted for one hour. The pre-test was performed one week before intervention and the post-test one week after the 8-week intervention. The research instruments included Nordic Musculoskeletal Questionnaire (NMQ), and the pain visual analog scale (VAS). Descriptive and inferential (Chi-square, Mann-Whitney, Wilcoxon, and McNemar test) statistics were used to analyze the data using SPSS software version 19.

**Results:**

the majority of the participants were males (67.2%) and in the age range of 60–74 years (82.8%). The mean age of samples in intervention and control groups was 68.45 (SD = 5.38) and 69.17 (SD = 5.86), respectively. The results showed that the prevalence of musculoskeletal disorders (MSDs) was decreased in the intervention group after the intervention (%Δ = -34.68, p < .05). The results also showed a statistically significant decrease in pain intensity of musculoskeletal in the intervention group, after the intervention (%Δ = -68.34, p < .001).

**Conclusions:**

Corrective exercises reduce the prevalence of MSDs and the pain intensity among older adults. It is recommended to pay attention to these exercises to improve physical health and reduce the prevalence of MSDs among older adults.

**Supplementary Information:**

The online version contains supplementary material available at 10.1186/s12891-023-06915-8.

## Background

Improvements in medical, surgical, and public health technologies have favorably increased human life expectancy [1]. Aging is a period that is associated with gradual, progressive, and spontaneous erosive changes in most organs and physiological functions of the body [2]. Changes occur in the function of musculoskeletal systems with age [3] and expose older adults to serious injuries such as bone fractures and long-term disabilities [4]. Aging is also a common cause of movement patterns that are inefficient and can lead to pain or injuries [5].

Musculoskeletal disorder (MSD) refer to any tissue damage in the musculoskeletal system that results in organ dysfunction [6]. These disorders may result in pain and loss of function and are among the most disabling and costly conditions in the United States [7]. Physical activity and exercise are considered one of the basic methods and primary care in dealing with chronic musculoskeletal pain, which, in addition to positive effects on the musculoskeletal system, reduces pain [8]. A review of previous literature confirms the positive effect of physical activity and exercise on the quality of life of the older. Moreover, physical activity to any extent can improve the health status of older adults [9].

In final WHO recommendations (2020 guidelines) on physical activity and sedentary behavior for all populations, mentioned that doing some physical activity is better than doing none. World Health Organization strongly recommended that as part of their weekly physical activity, older adults should do varied multicomponent physical activity that emphasizes functional balance and strength training at the moderate or greater intensity on 3 or more days a week [10]. Older adults generally suffer from motor restrictions due to osteoporosis and spine vertebrae deformities, so they must avoid performing intense exercises, as well as bending and rotating the trunk. Therefore, it is recommended to train these people to obtain and maintain the correct posture and guide them to drop wrong postural habits by performing corrective exercises that are not only simple to do but can also be implemented with minimum facilities and supervision at home by all people, including the older adults [11]. Corrective exercises are a branch of physical training that seeks to correct and eliminate various muscle, organic weaknesses, and anomalies and coordinate and balance movements using exact movements and programs and principles of exercise [12]. These exercises, to increase strength, endurance, and flexibility have been able to provide an acceptable effect [13]. Health & fitness professionals to address and fix movement compensations and imbalances use these exercises. These are applied to all clients, not just those recovering from injury [14]. On the other hand, residents at the nursing home are a different population from community-dwelling older people in ways that will likely influence their ability to participate in physical activity [15]. Repeatedly it has been shown that older adults in assisted living settings engage in very limited amounts of overall physical activity and particularly limited amounts of exercise [16–18]. Long periods of sedentary activity are common among older adults and harm both physical and mental health [18–20]. So far, research has been conducted to eliminate and reduce musculoskeletal disorders around the world [13]. There have been studies on the effect of various exercises such as strength, endurance, and functional exercises on the physical fitness of older adults [21–24]. The findings of the study by Mahmoudi et al. (2016) showed that corrective exercises could improve kyphosis and pain intensity among older adults [22]. The results of Mesquita et al.‘s study (2015) showed significantly better static and dynamic balance in the intervention group than in the control group [25].

Despite the previous studies, a review of the literature indicates that there are few studies on the effect of corrective exercises on MSDs among older adults and there is little knowledge about it. However, considering the increase in the number of older adults, it seems necessary to pay attention to the health status of this group and provide the necessary facilities to provide special services to the older adults, including exercise. Despite the conducted studies, more studies are still needed in this field to confirm the results of previous studies. On the other hand, considering the increase in the older adults’ population and the prevalence of chronic skeletal disorders in this age group, and assuming that corrective exercises can play an effective role in improving the condition and pain caused by musculoskeletal disorders in older adults, conducting interventions in this field is necessary. Therefore, the present study was conducted to determine the effect of corrective exercises on skeletal disorders among older adults living in nursing homes. The research hypotheses were:

1. There is a significant difference between the prevalence of musculoskeletal disorders in the intervention and control groups after the intervention (corrective exercises).
2. There is a significant difference between the pain intensity of musculoskeletal disorders after intervention in the intervention and control groups.

## Methods

This is a randomized controlled clinical trial study with pre-test and post-test design that was performed on older adults residing in a nursing home, in 2020.

### Study design and participants

The statistical population included all older adults residing in a nursing home (n = 397) in Rasht, the capital of Guilan province, located in the north of Iran. This city has only one non-profit, public nursing home that operates round the clock. This care center is located in a space of 17,000 square meters, to care for the older adults and disabled, in Suleiman Darab of Rasht. The main conditions for the clients’ admission are old age, lack of physical, motor, and spinal ability and, the inability of the family to take care of them. This center has wards 1, 2, and 3 for men and women, child and adolescent ward, and spinal cord lesion ward. In this research, the samples were selected from men’s and women’s wards.

**Fig. 1:**
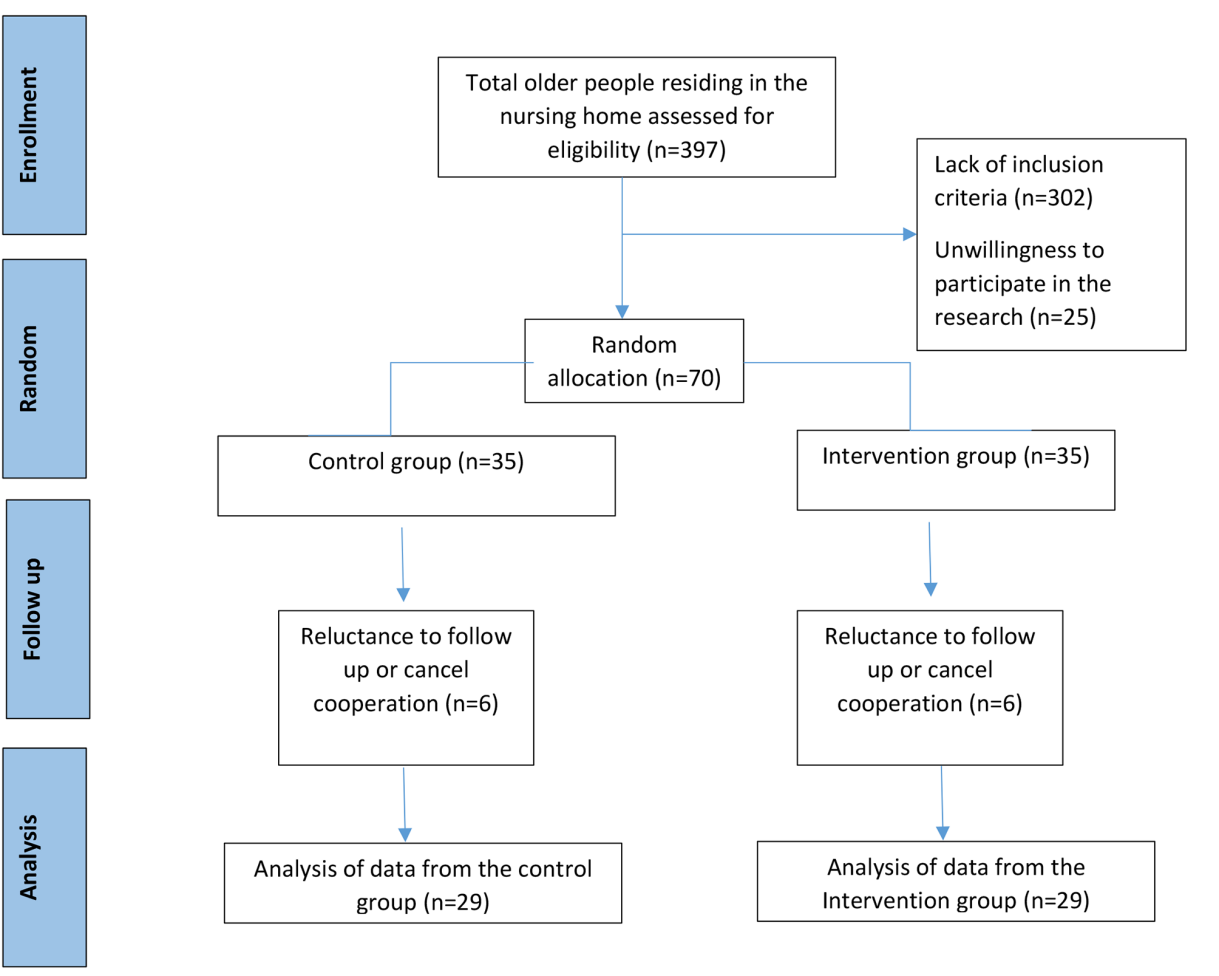
Flowchart of the allocation of participants to the study groups

Based on inclusion criteria, only the eligible older people were selected and randomly divided into intervention and control groups. A total of 70 eligible older people were selected and randomly divided into intervention (n = 35) and control (n = 35). In both groups (intervention and control), 6 subjects withdrew from cooperation due to unwillingness to continue cooperation. Finally, 29 samples remained in each group. The research flow is shown in Fig. 1.

Inclusion criteria included individuals aged 60 years and older, appropriate general health status (independence in daily activities and ability to perform training protocol), having a good state of cognition, vision and hearing, no history of orthopedic surgery in the past year, no musculoskeletal fractures in the last six months, no congenital anomalies and specific skeletal diseases such as rheumatoid arthritis and no cardiovascular disease or acute illnesses, lack of regular exercise and non-drug addiction and lack of painkiller and sedative use. Exclusion criteria also included mobility problems during exercise, not attending all training sessions, and the older adult’s unwillingness to participate in the study.

### Sampling method

A simple randomization method was used to select the subjects in the intervention and control groups. To this end, first, the names of the older were written on separate sheets, and then they were placed in an envelope. The participants were randomly placed in the intervention and control groups by taking them out of it one by one. At baseline, the researcher completed the questionnaires by asking questions about the samples. After training corrective exercises, the musculoskeletal status of the samples was checked again by the researcher. The pre-test was performed one week before and the post-test one week after the 8-week intervention (corrective exercise).

The sample size was calculated by considering the test power of 0.9 and the significance level of 0.05, 26 people for each group based on the following formula. Taking into account the probability of attrition, the final sample size of 35 people for each group was considered.

$$n=\frac{{\left({Z}_{1-\frac{\alpha }{2}}+{Z}_{1-\beta }\right)}^{2}({\delta }_{1}^{2}+{\delta }_{2}^{2})}{{({\mu }_{1}-{\mu }_{2})}^{2}}$$

$$n=\frac{{(1.96+1.28)}^{2}{(4.45}^{2}+{4.38}^{2})}{{\left(7-11\right)}^{2}}=25.57\cong 26$$

### Research instruments

Research instruments included a demographic information questionnaire (age, sex, marital status, number of children, number of grandchildren, previous place of residence, years of work history, income, medical history, body mass index (BMI), blood pressure, and pulse and respiration rate before and after the intervention), the Nordic Musculoskeletal Questionnaire (NMQ) and the pain Visual Analog Scale (VAS), which were completed by the researcher by asking relevant questions from the samples.

The Nordic Musculoskeletal Questionnaire is one of the most common questionnaires for determining musculoskeletal symptoms, presented by Kuorinka et al. at the Institute of Occupational Health in Nordic countries in 1987 [26]. It assesses only three areas of the body. Dawson later modified the instrument in 2009 and evaluated nine body parts [27, 28]. It is used to assess the patterns and prevalence of MSDs for the different parts of the body regions in the last 12 months [29]. This questionnaire consisted of general questions on the history of having trouble in any of the nine body regions: neck, upper back, lower back, shoulder, elbow, hand/wrist, hip, knee, and ankle/foot. This questionnaire was accompanied by a body map diagram, which facilitated the subjects to locate the pain or discomfort sites in their bodies. In addition, questions were also asked regarding the subject’s lifetime experiences, followed by the prevalent questions, and, lastly, on the items related to consequences of pain in the whole year. The response categories were restricted to “yes” and “no”. It is reliable for collecting information about the onset, prevalence, and consequences of musculoskeletal pain in the nine body regions [29]. This instrument has been used many times in epidemiological studies in Iran. The psychometric properties of this instrument were evaluated by Mokhtarinia et al. (2015). The range of the kappa coefficient was calculated between 0.78 and 1 [28].

In the present study, the pain Visual Analog Scale (VAS) was used to assess pain intensity. This scale is one of the oldest, easiest, and best-validated measures to assess pain [30]. The VAS is a 10 cm line with anchor statements ranging from zero (no pain) on the left to 10 (the most severe) on the right. Mild, moderate, and severe pain are indicated by scores 1–3, 4–7, and 8–10, respectively [31, 32]. Its validity and reliability are excellent and its reliability was confirmed in Iran with a correlation coefficient of r = .88 [32].

### Intervention

The exercise class started with 10-min warm-up exercises (including stretching and balance exercises), and then continued for 30–40 min performing corrective exercises after the researcher in groups and individually, according to the conditions of each sample. At the end of the training class, cooling exercises and returning to the initial state were carried out for about 10 min. The intervention group performed corrective exercises for 8 weeks and 3 sessions per week and each session lasted for one hour. Exercises were designed based on each person’s ability and the principles governing the exercise, including the exercise intensity, gradual increase in exercise intensity, exercise duration, the principle of load progression, and the movement pattern of the exercise [12]. No special exercises were considered for the control group, and they performed daily and routine activities. Each stretching exercise was performed slowly and in a controlled manner. Increased resistance training was used to increase muscle strength. According to the principle of load progression, each session was added to the number of repetitions so that as subjects continued the exercises, they performed them more frequently, within a shorter rest time, and without feeling tired. The exercises started with 10 repetitions and reached 30 times at the end of the eighth week. Exercises include eight types of physical activity (exercises for the back, neck, shoulders, knees, ankles and feet, pelvis and abdomen, elbows, wrists, and hands). The exercises were designed from simple to complex. Special exercises were considered for each of the above-mentioned organs separately. The training program for the neck was of the type of isometric exercises. The special exercise program for the lower back was flexion exercises with an emphasis on strengthening the abdominal muscles and back extensors. The shoulder training program emphasized muscle strengthening and flexibility. Strengthening and flexibility exercises were also considered for the hands. More details about the intervention protocol are presented in Appendix 1.

All the exercises were done by one of the researchers and under the supervision of a physiotherapy specialist. Participants were asked not to perform any additional exercise. The control group was asked to do their normal daily activities and not participate in any exercise program. After completing the study, the control group will undergo the exercise intervention protocol.

### Treatment fidelity

Treatment fidelity refers to the methodological strategies used to monitor and enhance the reliability and validity of behavioral interventions [33]. The US National Institute of Health (NIH) Behaviour Change Consortium has proposed a model of fidelity which identifies 5 areas in which fidelity should be addressed in behaviour change research: study design, the training of intervention providers, and treatment delivery, receipt, and enactment [34].

To comply with treatment fidelity, in designing the study, the intervention (corrective exercises) was designed by the research team based on the book by Hertling et al. [35]. One of the researchers (Master of Geriatric Nursing) was trained under the supervision of a physiotherapy specialist, and he provided the intervention. He was present at the nursing home at specific times during the weekdays and performed exercises for the sample under the supervision of an ergo therapist at a nursing home. He taught the exercises to the participants under his supervision. He had full control over how the samples performed the intervention and how the participants performed the exercises. After training the samples in the researcher’s presence according to the location of the disorder, corrective exercises were performed. Educational pamphlets were also used by older adults to better understand exercises. Thus, using training pamphlets, how to do the exercises was taught visually for the samples. The training was performed on the bed of each older adult individually or in groups according to the musculoskeletal symptoms of each sample. Older adults with the same musculoskeletal symptoms were grouped and trained. For example, older adults with musculoskeletal symptoms in the neck were grouped and performed corrective exercises as a group. The educational content and intervention method were the same for all samples with similar musculoskeletal symptoms. Furthermore, the number of exercises and the duration of the intervention were the same for all samples with similar musculoskeletal symptoms. The frequency and timing of interventions were monitored continuously using auxiliary tools such as an hourglass and counting tools such as line markers and finger counting.

### Data Analysis

Data analysis was performed using descriptive and inferential statistics by Statistical Package for the Social Sciences version 19 (SPSS; IBM, Armonk, NY, USA). Normality was measured by the Kolmogorov-Smirnov test. The data were not normally distributed, so non-parametric tests were used for data analysis. To evaluate the effect of the intervention on MSDs, the post-intervention results were evaluated using the Chi-square test. To evaluate the effect of the intervention on pain intensity, the post-intervention results and the mean score of pain intensity of musculoskeletal disorders were compared between the two groups compared using the Mann-Whitney test. To compare pain variables before and after the intervention, the Wilcoxon test was used. The McNemar’s test was used for the presence or absence of MSDs.

The percentage of change pre- and post-test was calculated as.

Percentage change from pretest to posttest =$\frac{\text{p}\text{o}\text{s}\text{t}\text{t}\text{e}\text{s}\text{t}\ \text{s}\text{c}\text{o}\text{r}\text{e}-\text{p}\text{r}\text{e}\text{t}\text{e}\text{s}\text{t}\ \text{s}\text{c}\text{o}\text{r}\text{e}}{\text{p}\text{r}\text{e}\text{t}\text{e}\text{s}\text{t}\ \text{s}\text{c}\text{o}\text{r}\text{e}}\times 100$

The Chi-square or Fisher’s exact test was used to evaluate the homogeneity of background (demographic) variables in the two intervention and control groups. All calculations were performed at a significant level of P < .05.

### Ethical consideration

This study was performed under the ethical standards as laid down in the Declaration of Helsinki and its later amendments or comparable ethical standards. Written informed consent was obtained from all individual participants included in the study. For illiterate participants, informed consent was obtained from their literate legally authorized representatives/guardians.

## Results

Results on demographic characteristics showed that the majority of the participants were males (67.2%) and in the age range of 60–74 years (82.8%). The mean age of older adults in intervention and control groups was 68.45 (SD = 5.38) and 69.17 (SD = 5.86), respectively. Table 1 shows the frequency distribution of demographic characteristics of older adults.

**Table 1:** Frequency distribution of demographic characteristics of the elderly residing in nursing homes in the intervention and control group

Group Variable		Intervention		Control		Total		Statistical estimation
		Number	Percentage	Number	Percentage	Number	Percentage	
Age	60–74 years old	26	89.65	22	75.86	48	82.8	P = .594 X2 = 16.93
	75–90 years old	3	10.34	7	24.14	10	17.2	
	Mean (SD)	68.45 (0.38)		69.17 (5.86)		68.81 (5.59)		
Sex	Female	10	34.5	9	31	19	32.8	P = .780 X2 = 0.078
	Male	19	65.5	20	19	39	67.2	
Education	Illiterate	11	37.9	7	24.1	18	31	P = .259 F = 3.960
	Primary	14	48.3	12	41.4	26	44.8	
	High school	2	6.9	7	24.1	9	15.5	
	University	2	6.9	3	10.3	5	8.6	
Marital status	Single	12	41.4	12	41.4	24	41.4	P = 1.000 X2 = 0.000
	The wife has died	15	51.7	15	51.7	30	51.7	
	Divorced	2	6.9	2	6.9	4	6.9	
Previous job	Farmer	11	37.9	13	44.8	24	41.4	P = .611 F = 1.957
	Retired	7	24.1	8	27.6	15	25.9	
	seller	4	13.8	5	17.2	9	15.5	
	Hand seller	7	24.1	3	10.3	10	17.2	

Results of comparing MSDs in the intervention and control groups before and after intervention are shown in Table 2.

**Table 2:** Percentage of change in musculoskeletal disorders of the elderly in the intervention and control groups before and after the intervention

Group Variable			Intervention	Control	P-value	
Musculoskeletal disorders (%)	Before intervention		34.54	32.02	Neck	P=.607
					Pelvis	P = .196
					Left knee	P = 1.000
	After intervention		22.65	31.22	Neck	P = .004**
					Pelvis	P = .596
					Left knee	P = .097
Percentage of changes in musculoskeletal disorders (%)^a^			-34.42	2.49		
P-value		Neck	P = .000**	P = 1.000		
		Pelvis	P = .016*	P = 1.000		
		Left knee	P = .002**	P = 1.000		

Note. ^a^ Percentage change from pretest to posttest =$\frac{posttest score-pretest score}{pretest score}\times 100$

P-value = Statistical estimation; *p < .05, **p < .001

The above table shows that the prevalence of MSDs after the intervention decreased by 11.89% in the intervention group, which was statistically significant in some areas (neck (P = .000), pelvis (P = .016), and left knee (P = .002)) (%Δ = -34.68, p < .05). However, the prevalence of MSDs after the intervention in the control group was not significant (P = 1.000). Since there was a significant difference in the prevalence of MSD in the neck, left knee, and pelvis areas before and after the intervention, for this reason, the results of the above table focus on the analysis of the results in these three areas.

The results regarding the frequency distribution and comparison of the pain intensity of musculoskeletal disorders in the intervention and control groups before and after the intervention are shown in Table 3.

**Table 3:** Frequency distribution and comparison of pain intensity of musculoskeletal disorders in the intervention and control groups before and after the intervention

Pain intensity of musculoskeletal disorders		Intervention Group (n = 29)		Control Group (n = 29)		Between group ^a^
		Number (%)	Mean (SD)	Number (%)	Mean (SD)	
Before intervention	Mild pain (1–3)	13(44.8)	3.38 (1.22)	17 (58.6)	3.34 (1.17)	P = .733 Mean difference = 0.49
	Moderate pain (4–7)	16 (55.2)		12 (41.4)		
After intervention	Mild pain (1–3)	28(96.6)	1.07 (0.923)	17 (58.6)	3.34 (1.17)	P = .000** Mean difference = -2.27
	Moderate pain (4–7)	1 (3.4)		12 (41.4)		
Within group ^b^		P = .000 Mean difference = -2.76		P = 1.000 Mean difference = 0		

Note. ^a^ Mann-Whitney U Test, ^b^ Wilcoxon Test

*p < .05, **p < .001

The above table shows that, significantly, the mean score of pain intensity in the intervention group has decreased after the intervention (%Δ = -68.34, p < .001).

## Discussion

This study aimed to determine the effect of corrective exercise on MSDs among older adults residing in nursing homes. The results showed that the prevalence of MSDs after the intervention decreased by 11.89% in the intervention group, which was statistically significant in some areas (neck, pelvis, and left knee). However, the prevalence of MSDs in the control group was not significant. The results of a study by Nawrocka et al. (2019) conducted to assess the relationship between objectively measured physical activity and perceived work ability and musculoskeletal disorders among adult, middle-aged, and older women, and reported significant relations between the frequency of occurrence of musculoskeletal problems and meeting physical activity recommendations in women aged 50–64 years. In this study, both the prevalence of musculoskeletal problems reported in the past year and the intensity of musculoskeletal pain during last the week were significantly lower in women who met physical activity recommendations [36]. The results of a study by Safari bak et al. (2017) indicated that eight weeks of selected exercise could significantly improve all balance indexes in older patients with Knee Osteoarthritis [37]. Another study investigated to evaluate the effectiveness of a home-based exercise intervention (HBEI) to reduce Knee osteoarthritis (KOA) symptoms and improve the physical functioning of older patients. The results showed that HBEI might be effective in relieving KOA symptoms and increasing physical functioning in older patients [38].

In the present study, results also showed that moderate musculoskeletal pain in the intervention group turned into mild pain after the intervention in 51.8% of cases, which was statistically significant. In other words, the corrective exercise program reduced pain levels in the participants. This result is identical to that reported in a previous study, which investigated the effect of isometric exercise on back pain in older persons and reported a significant decrease in back pain after the exercise program [39]. The results of a study by Niederstrasser et al. (2022) indicated that mild, moderate, and high physical activity was associated with a lower likelihood of suffering from musculoskeletal pain compared to being sedentary [40]. Another study investigated to evaluate the clinical effectiveness of a supervised neuromuscular exercise programme in older people with chronic musculoskeletal pain and reported this exercise has the potential to reduce pain and improve self-efficacy and physical function in older people with chronic musculoskeletal pain [41]. Mahmoudi et al. (2017) showed that the pain intensity of MSDs decreased by 2.48 in the intervention group but increased by 0.11 in the control group [22]. Cruz et al. (2016) found that Pilates exercises reduced lower-back pain in adults and the older [42]. The results of the above-mentioned studies show that steady exercise relieves musculoskeletal pain. These findings provide insights that may inform interventions aimed at reducing the risk of developing frequent musculoskeletal pain complaints [40]. Therefore, the development and introduction of suitable exercise programs will contribute to health [5].

In general, exercise effectively maintains physical fitness and helps maintain a healthy weight, healthy bone density, muscle strength, and joint mobility, improves physiological health, reduces the risk of surgery, and strengthens the immune system. Exercise causes more blood to flow to the musculoskeletal system [43]. In addition to being simple, corrective exercises can be easily used at home and by everyone, including the older, with minimum facilities and supervision. The exercises used in the aforementioned articles, which also had high effectiveness, were not completely safe and increased the risk of fractures among older with fragile bones; therefore, they require a lot of supervision and guidance [22]. Exercise also stimulates the production of pain-suppressing hormones and plays a preventive or reducing role in the onset of pain by increasing the pain threshold. Since the beneficial effects of exercise programs on people’s health are quite clear and considering the relevant literature, which shows the positive effects of these programs, it is essential to pay a lot of attention to exercise to improve physical health and reduce the prevalence of MSDs [43].

Physical activity is essential for healthy aging and offers many health benefits, including a reduced risk of chronic diseases and premature death [44]. Physical activity, rehabilitation, or exercise may improve independence and prevent the decline in ADL in older residents in long-term care facilities [45–47]. Moreover, the implementation of a physical activity program improves physical and cognitive functions, increases the quality of life, and decreases depressive symptoms in older adults [44]. The amount of physical activity is one of the determinants of the maintenance of independence in older adults [44]. However, older adults are physically inactive and reluctant to join social activities [48]. This situation is more evident in nursing home residents and is partly attributed to the limited space, fewer exercise coaches, and reduced physical function [44]. To help support populations to achieve the targets and maintain healthy levels of physical activity, all countries are advised to develop and implement appropriate national and subnational policies and programmes to enable people of all ages and abilities to be physically active and improve their health [49]. Physical activity recommendations for older adults are a minimum of 150 min of moderate-intensity aerobic physical activity or at least 75 min of vigorous-intensity aerobic physical activity or an equivalent combination of both throughout the week [50]. It is thus important that residential aged care providers offer opportunities to experience exercise to all residents [51]. Nursing homes should strive to develop meaningful activities for residents to occupy their time and to provide residents with a meaningful sense of purpose [52]. Efforts should be made to develop exercise health education programs for older adults and empower them to engage in suitable daily exercises such as active games in a safe environment. This will enable them to have positive experiences with exercise that improve their physical activity self-efficacy [44]. Future interventions should account for the care home context. Moreover, proper educational programs for physical activity engagement interventions, adequate facilities, and personnel to provide exercise guidance for nursing home residents are needed [44].

Our study has several limitations. One of the limitations of this study was the impossibility of precise control of biological factors such as diet and sleep. For example, even though the daily diet of the nursing home was the same, the possibility of standardizing the nutritional status was out of the researcher’s control due to the different tastes and appetites of the subjects. Another limitation of this research is that it was done without blinding. Because blinding was not possible due to the nature of the intervention. However, the risk of bias due to lack of blinding was carefully considered. The participants in the study were informed about the objectives of the study and possible risks. On the other hand, participation in the study was voluntary and the samples were informed about the allocation to the studied groups. In addition, participants in the control group were assured that after the completion of the current research, the exercises will be taught and performed for them if they wish. Moreover, small sample size, a single-center study, short-term follow-up, lack of control over muscle strength, type, severity, and the number of musculoskeletal limitations and mental status of subjects such as level of physical fitness, level of motivation, personality type, psychological complications caused by family and economic problems that could affect the generalizability of the results was the other limitations of the present study.

## Conclusion

According to the findings of the present study, it can be said that corrective exercises can reduce the prevalence of MSDs and the subsequent pain intensity among older adults. Physical activity and exercise could be non-pharmacological strategies for helping people with musculoskeletal disorders. The results of this study can be considered by the officials and managers of medical centers and nursing homes to plan and provide the necessary arrangements for corrective exercise, to reduce musculoskeletal pain and subsequently improve health and increase the quality of life in older adults. Nurses and health care providers can include corrective exercises in the care program for older adults with musculoskeletal disorders.

### Electronic supplementary material

Below is the link to the electronic supplementary material.

Appendix 1

## Data Availability

All the data cannot be publicly shared because of ethical concerns. The datasets without any confidential information used and/or analyzed during the current study are available from the corresponding author at reasonable request.
