# Supplementary material for: The effect of corrective exercises on musculoskeletal disorders among the older adults residing in a nursing home in Rasht, Guilan, Iran
Source: BMC Musculoskelet Disord. 2023 Oct 17;24:820. doi: 10.1186/s12891-023-06915-8 (PMC10580666; doi:10.1186/s12891-023-06915-8)
Supplement: Supplementary file 1 — Appendix 1 [file 12891_2023_6915_MOESM1_ESM.docx]

**Appendix 1**

**Trial protocol**

The intervention (corrective exercises) in this study was designed by the research team based on the book by Hertling et al. One of the researchers (Master of Geriatric Nursing) was trained under the supervision of a physiotherapy specialist, and he provided the intervention. He was present at the nursing home at specific times during the weekdays and performed exercises for the sample under the supervision of an ergotherapist at a nursing home. He taught the exercises to the participants under his supervision. He had full control over how the samples performed the intervention and how the participants performed the exercises. After training the samples in the researcher's presence according to the location of the disorder, corrective exercises were performed. Educational pamphlets were also used by older adults to better understand exercises. Thus, using training pamphlets, how to do the exercises was taught visually for the samples. The training was performed on the bed of each older adult individually or in groups according to the musculoskeletal symptoms of each sample. Older adults with the same musculoskeletal symptoms were grouped and trained. For example, older adults with musculoskeletal symptoms in the neck were grouped and performed corrective exercises as a group. The educational content and intervention method were the same for all samples with similar musculoskeletal symptoms. Furthermore, the number of exercises and the duration of the intervention were the same for all samples with similar musculoskeletal symptoms. The frequency and timing of interventions were monitored continuously using auxiliary tools such as an hourglass and counting tools such as line markers and finger counting.

The exercise class started with 10-min warm-up exercises (including stretching and balance exercises), and then continued for 30-40 minutes performing corrective exercises after the researcher in groups and individually according to the conditions of each sample. At the end of the training class, cooling exercises and returning to the initial state were carried out for about 10 minutes. The intervention group performed corrective exercises for 8 weeks and 3 sessions per week and each session lasted for one hour. Exercises were designed based on each person's ability and the principles governing the exercise, including the exercise intensity, gradual increase in exercise intensity, exercise duration, the principle of load progression, and the movement pattern of the exercise. No special exercises were considered for the control group, and they performed daily and routine activities. Each stretching exercise was performed slowly and in a controlled manner. Increased resistance training was used to increase muscle strength. According to the principle of load progression, each session was added to the number of repetitions so that as subjects continued the exercises, they performed them more frequently, within shorter rest time, and without feeling tired. The exercises started with 10 repetitions and reached 30 times at the end of the eighth week. Exercises included eight types of physical activity (exercises for the back, neck, shoulders, knees, ankles and feet, pelvis and abdomen, elbows, wrists, and hands) according to the example images below.


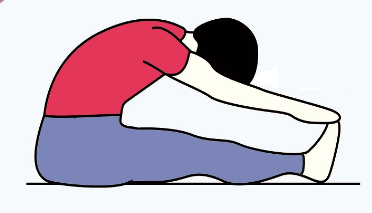


(1)


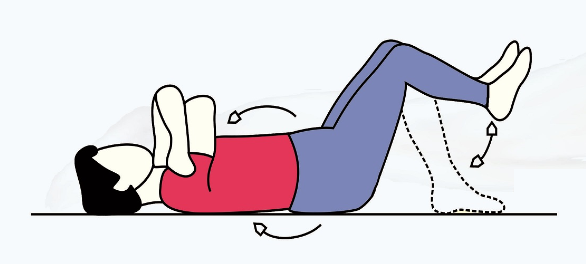


(2)


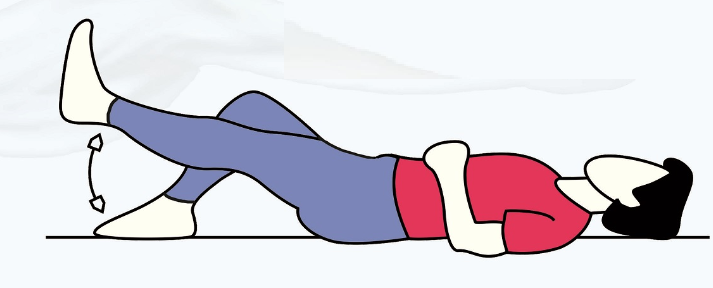


(3)


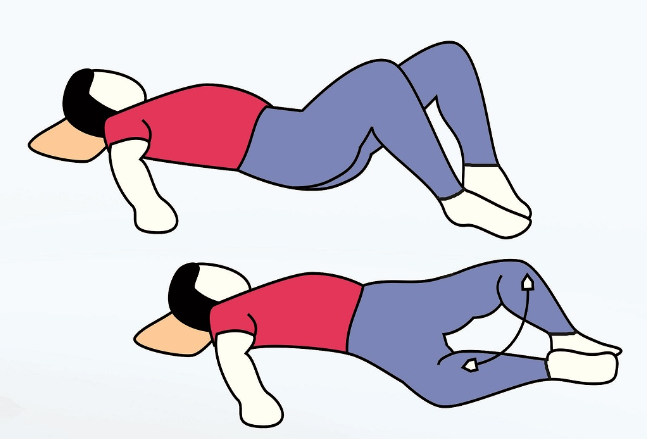


(4)


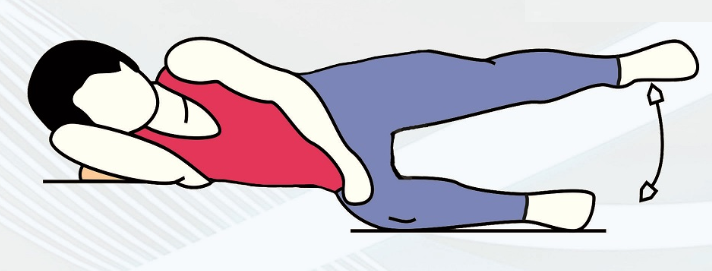


(5)


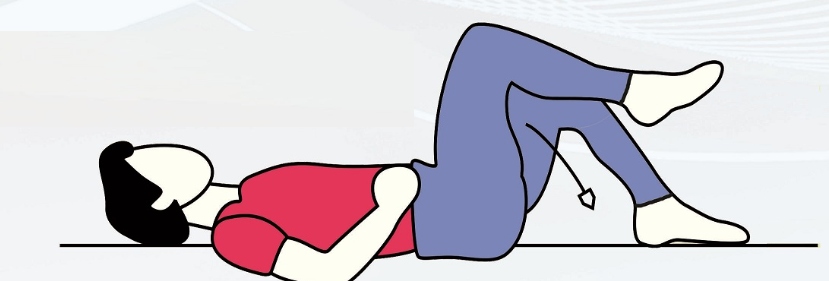


(6)


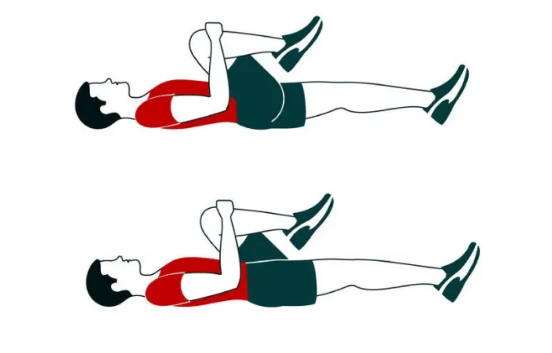


(7)


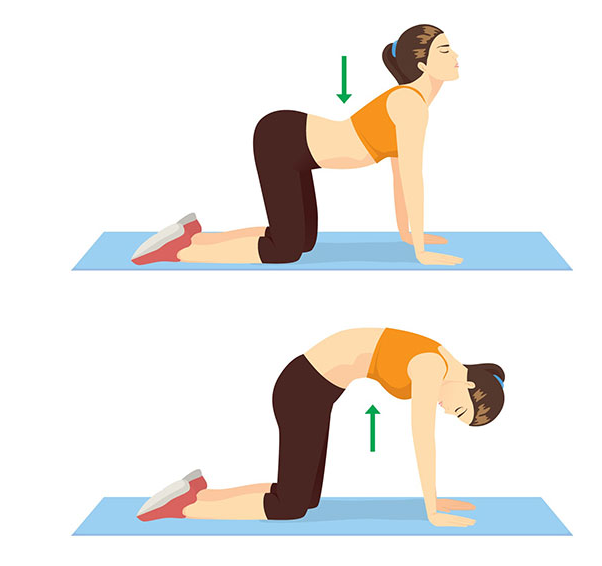


(8)


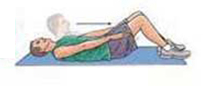


(9)


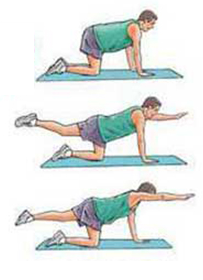


(10)


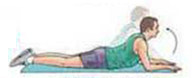


(11)


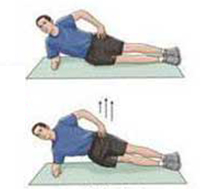


(12)


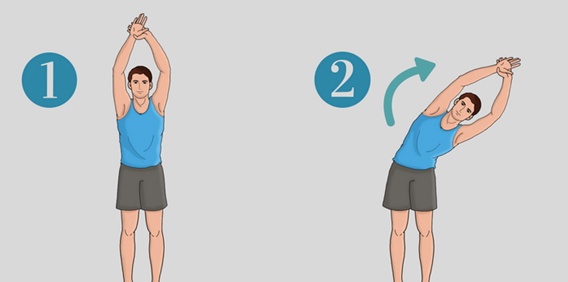


(13)


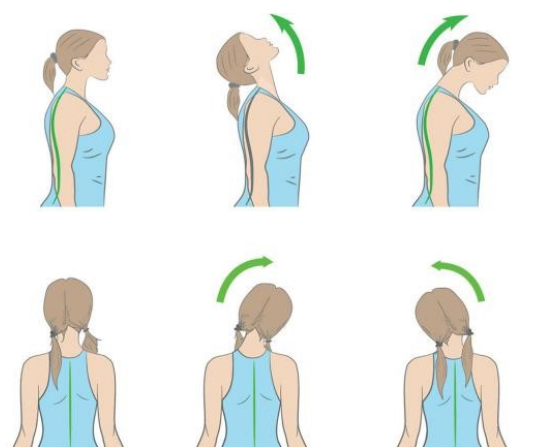


(14)
